# Breastfeeding, first-food systems and corporate power: a case study on the market and political practices of the transnational baby food industry in Brazil

**DOI:** 10.1186/s12992-024-01016-0

**Published:** 2024-02-06

**Authors:** Cindy Alejandra Pachón Robles, Mélissa Mialon, Laís Amaral Mais, Daniela Neri, Kimielle Cristina Silva, Phillip Baker

**Affiliations:** 1 Corporación Universitara Remington, Facultad de la salud, Grupo de Neurociencias y Envejecimiento, Medellín, Colombia; 2 Trinity College Dublin, Dublin, Ireland; 3 Brazilian Institute for Consumer Defense (Idec), São Paulo, Brazil; 4 Center for Epidemiological Research in Nutrition and Health (Nupens), University of São Paulo (USP), São Paulo, Brazil; 5 Institute of Social Medicine (IMS), University of the State of Rio de Janeiro (UERJ), Rio de Janeiro, Brazil; 6 Institute for Physical Activity and Nutrition, Deakin University, Geelong, Australia

**Keywords:** Commercial milk formula, Breastmilk substitutes, Commercial determinants of health, Infant and young child feeding, Child and maternal health

## Abstract

**Background:**

The exploitative marketing of commercial milk formula (CMF) reduces breastfeeding, and harms child and maternal health globally. Yet forty years after the International Code of Marketing of Breast-Milk Substitutes (The Code) was adopted by WHO member states, many countries are still to fully implement its provisions into national law. Furthermore, despite The Code, worldwide CMF markets have markedly expanded. In this paper, we adopt Brazil as a case study to understand the power of the baby food industry’s marketing and corporate political activity, and how this influences the country’s ‘first-food system’ in ways that promote and sustain CMF consumption.

**Methods:**

We used a case study design, drawing data from from documents and key informant interviews (*N* = 10).

**Results:**

Breastfeeding rates plummeted in Brazil to a historic low in the 1970s. A resurgence in breastfeeding from the mid-1980s onwards reflected strengthening political commitment for a national policy framework and breastfeeding protection law, resulting in-turn, from collective actions by breastfeeding coalitions, advocates, and mothers. Yet more recently, improvements in breastfeeding have plateaued in Brazil, while the industry grew CMF sales in Brazil by 750% between 2006 and 20. As regulations tightened, the industry has more aggressively promoted CMF for older infants and young children, as well as specialised formulas. The baby food industry is empowered through association with powerful industry groups, and employs lobbyists with good access to policymakers. The industry has captured the pediatric profession in Brazil through its long-standing association with the Brazilian Society of Pediatrics.

**Conclusion:**

Brazil illustrates how the baby food industry uses marketing and political activity to promote and sustain CMF markets, to the detriment of breastfeeding. Our results demonstrate that this industry requires much greater scrutiny by regulators.

**Supplementary Information:**

The online version contains supplementary material available at 10.1186/s12992-024-01016-0.

## Introduction

The commercial determinants of health (CDOH) are receiving growing attention from researchers, advocates and policymakers, with the purpose of informing societal responses to so-called ‘industrial’ or ‘manufactured’ epidemics, resulting from harmful commercial products, corporate practices and systems [1]. In this paper, we focus on the commercial determinants of infant (aged ≤ 12 months), young child (12–36 months) and maternal health, by examining the marketing and political activities of the baby food industry, and resistance by public health actors to protect breastfeeding and public health in Brazil. This builds upon a programme of research, involving various other country case studies [2–6].

To promote optimal growth, development and health, the World Health Organization (WHO) recommends infants initiate breastfeeding in the first hour of life, are then exclusively breastfed for six months, and thereafter receive nutritious and safe complementary foods, while breastfeeding continues for up to two years of age or beyond [7]. Yet, less than half of the world’s children meet these three recommendations, denying them the right to the best possible nutrition and health breastfeeding provides [8]. One powerful factor impeding global progress on breastfeeding, is the exploitative marketing and promotion of breastmilk substitutes (BMS) [8–11]. Exposure to such marketing results in reduced breastfeeding initiation, exclusivity and duration, irrespective of country context [8]. Baby food corporations use sophisticated marketing techniques, including new forms of digital marketing, to influence mothers, caregivers and families to expand their markets [8, 12]. Commercial BMS are foods marketed or otherwise represented as partial or total replacements for breastmilk, including those for children aged 0–36 months, as well as supplements and feeding equipment such as bottles, pacifiers and teats. The main type of BMS marketed worldwide are commercial milk formulas (CMF), including standard (for 0–6 months), follow-on (7-12 m), toddler (13-36 m) and specialized (or ‘therapeutic milks’) product categories [13]. The WHO and United Nations Children’s Fund (UNICEF) *Global Strategy on Infant and Young Child Feeding* calls on governments to adopt policies that protect, promote and support breastfeeding, This includes the adoption of the *The International Code of Marketing of Breast-Milk Substitutes* (1981) and subsequent World Health Assembly resolutions (*The Code*) into national law [7]. The adotion fo *The Code* by WHO member states was a remarkable achievement at the time, given concerted resistance from the baby food industry as well as the United States (US) and other dairy-producing countries [2, 14]. Since then, civil society organizations, international agencies and experts have worked with governments to implement *The Code*. Yet forty years later there is still a long way to go. As of 2020, 136 of 194 reporting countries (70%) have adopted at least some provisions of The Code into national law, but just 35 (18%) have adopted all provisions, and 58 (30%) have no legal measures whatsoever [15]. Furthermore, global CMF sales grew 36-fold between 1978 and 2019, from US$1·5 billion to $55·6 billion annually, reflecting a situation where more infants and young children are fed CMF than ever before [2, 6, 8]. Elsewhere we describe this transition to higher formula diets as reflecting transformations in ‘first-food systems’, defined as the food systems that provision foods for infants and young children, and that structure feeding practices at the population level [12].

Recent studies demonstrate how a small number of transnational corporations headqaurtered in the European Union and United States, especially Nestlé, Danone, Reckitt Benckiser (Mead Johnson), Abbott Laboratories, and Friesland Campina – have powerfully shaped first-food systems in ways that denormalise breastfeeding, and that drive the expansion of CMF in the diets of infants and young children on a global scale. Recent studies report how these companies and affiliated industry groups engage in corporate political activity (CPA) to counteract regulatory threats, and to foster favourable policy, regulatory and knowledge environments that enable their marketing and continuing market expansion [2–6, 16, 17]. This CPA includes, for example, the shaping of science by funding and disseminating corporate research, influencing public opinion through public relations initiatives, and lobbying governments through a coordinated international network of front groups to counter regulatory threats [2, 6]. It is crucial that such practices are documented, given such activities are a major barrier to implementing *The Code* into national law, and to advancing the rights and interests of breastfeeding women, infants and young children everywhere [6, 8].

In this paper we adopt Brazil as a case study to illustrate the ongoing worldwide struggle to protect breastfeeding and public health from harmful commercial practices. The country has among the world’s strongest national breastfeeding protection laws [18], the *Brazilian Standard for the Marketing of Food for Infants and Early Childhood Children, Nipples, Pacifiers and Bottles* (*Norma Brasileira de Comercialização de Alimentos para Lactentes e Crianças de Primeira Infância, Bicos, Chupetas e Mamadeiras* or NBCAL in Portuguese, or *The Brazilian Code*). And yet, Brazil has also experienced rapid CMF sales growth, and represents a major market for the industry in Latin America. Our aim in this study is to examine the market and political practices used by the industry to shape Brazil’s first-food system in its commercial interests, in ways that undermine breastfeeding and promote the growth of the country’s CMF market. Our intention is to inform new public health action to advance breastfeeding. We first examine the fall and then resurgence of breastfeeding in the country in relation to historical first-food systems change, then examine the baby food industrys marketing and CPA. We further investigate the activities of civil society groups and coalitions, and policymakers, in resisting the power of this industry, and its collective efforts to protect, promote and support breastfeeding.

## Methods

Given the complex and multi-variable nature of the topic under study, we adopted a theoretically guided case study design [19], and process tracing method [20, 21]. This involved several steps. First, describing the scope and setting of the case study; second, collecting data from documentary sources and key informant interviews; and finally, synthesising results. We did not place constraints on the time-period under study but allowed for an emerging understanding of historical events. CAPR, who had working proficiency in Brazilian Portuguese, led the data collection and analysis under the supervision of MM (working proficiency in Brazilian Portuguese), with support and guidance from PB (native English-speaker) and LAM (native Brazilian). Data collection and analysis took place between January and August 2020 and was updated in June-July 2021 and in February 2023. DN, LAM and KCS substantially contributed to the interpretation of the results. CAPR, PB and MM wrote the manuscript, with significant contributions from DN, LAM and KCS.

### Scope and setting of the case study

Brazil is the largest country in Latin America with a population of 214 million. It is an upper-middle income country with a gross national income of US$7,740 per capita in 2021 [22]. Brazil is a federal presidential republic comprising a president with executive powers; a bicameral National Congress of Brazil comprising a Chamber of Deputies and a Senate, with legislative powers; twenty-six state governments and one federal district government; and a Supreme Federal Court, various Superior Courts, a National Justice Council and Regional Federal Courts. Links with the international system include membership in the United Nations (UN) including the WHO (1945), the World Trade Organization (WTO) (1995), the Southern Common Market (MERCOSUR) (1991), G20 and BRICS (Brazil, Russia, India, China and South Africa) [23]. Brazil regularly participates in multilateral health fora, including the WHO’s main governing bodies and initiatives, and at times, has been recognised for its global health leadership role [24, 25].

Brazil is a signatory to 14 UN human rights treaties, including the Convention on the Rights of the Child and the Convention on the Elimination of All Forms of Discrimination Against Women [26]. Brazil comprises diverse ethnic groups and religious affiliations, reflecting its ancient Indigenous history and peoples, and colonial and post-colonial era migration. From the 16th Century Brazil was a colony of the Portuguese empire, until its independence in 1822. From 1964 to 1985, an authoritarian military junta was in power. Following this, democratic government and neoliberal economic reforms under the Collor and Cardoso administrations, resulted in rapid increases in socio-economic inequality. Following the election of the leftist Lula administration in 2003, the country underwent major social policy reforms, that contributed to significant reductions in malnutrition and poverty. Under the recent right-wing Bolsonaro administration, many of these reforms were challenged or even reversed.

### Data collection

#### Documents

To help develop initial concepts, guide our data collection and organize the results, we were orientated by theoretical frameworks developed in earlier studies on first-food systems and corporate power [2]. We followed the method developed by Mialon et al. to source documents on the CPA of the food industry [27].

#### Documentary evidence

Data collection first consisted of focused searches of academic and internet databases using diverse key words and search strings derived from the framework. As our understanding of the case study evolved, we conducted further branching searches to source additional documents. This also included more specific searches on the two market leaders Nestlé and its Foundation (Fundação Nestlé Brazil, in Portuguese) and Danone, including a search of their national corporate websites. We further collected publicly available documents from the websites of key actors in Brazil’s first-food system, including government agencies, professional associations, civil society organizations, the media, academia and wider industry. We identified these actors through consultations with local experts and from documents (Supplemental material, Table S1).

#### Semi-structured key informant interviews

We triangulated the documentary data with key informant interviews. Participants were recruited using a purposive snowball sampling method [28]. We initially contacted 35 individuals by email. Eight individuals declined to participate, and seventeen, including four from baby food companies, did not respond. In total, we conducted 10 interviews in Portuguese with individuals from government agencies (*n* = 3), civil society organizations (*n* = 4) and academia (*n* = 3), all of whom had expertise in public health nutrition, and specifically infant and young child feeding. Of these, four interviews were conducted in person, and the remainder by online video call, using Skype and Zoom software. The interviews were semi-structured and we used an interview guide developed and pilot-tested by PB in other countries. Interviews were recorded and notes were taken with prior consent. We conducted interviews until no other participants agreed to participate, within our timeframe for data collection for our study. Interviews were transcribed by CAPR and de-identified.

*COVID-19 and data collection*: We undertook the study during the early stages of the COVID-19 pandemic, which incurred logistical changes with recruitment, prevented in-person interviews, and created challenges for the first author who conducted the interviews. Professionals working in the health sector, for example, were occupied with other more urgent priorities. The pandemic therefore impacted our data collection and delayed our analysis and writing.

### Data analysis and reporting

Data analysis proceeded through several steps. First, the translation of quotes from Portuguese to English was undertaken by MM and reviewed by PB. Second, the documentary and interview data were coded, guided by our initial framework, and modified in an iteractive process as key themes emerged, alongside discussions among the team of researchers. A random sample of 10% of the documentary data and 10% of the interview data was reviewed by LAM and DN respectively, and then reviewed again by MM.

## Results

We presented our findings first as a timeline of key events in Brazils’ evolving first-food systems; then provide an overview of how key corporate actors and interest groups have influenced Brazil’s first-food system, beginning with the market and political practices of the baby food industry, followed by the role of government and civil society groups.

### Brazil’s evolving first-food system

We identified three major periods of historical change regarding first-food system in Brazil. These dynamics are attributed to changes in multiple factors that have structured feeding practices across the Brazilian population. The three periods are summarized below:

1. **1940-1980 s – period of breastfeeding decline**: In 1940, 96.0% of Brazilian infants were exclusively breastfed at one month of age. By 1974, this figure was just 39.2% [29]. The median duration of breastfeeding reached its lowest point of just 2.5 months in 1974-75 [30]. Factors explaining this transition likely included income growth, urbanization, rising women’s work outside of the home, the growing use of pacifiers, the increased medicalization of pregnancy and birth, and more intensive marketing practices of the baby food industry [29, 31, 32].
2. **1980s-mid-2000s – the resurgence of breastfeeding**: The median duration of breastfeeding increased from 2.5 to 6.8 months by 1986, 9.9 months by 1999 [30], and 14 months in 2006-7 [33]. Between 1986 and 2006, the prevalence of exclusive breastfeeding under six months increased from 2.9 to 37.1%; and continued breastfeeding at one year of age (12–14 months) from 25.5 to 47.2% [34]. This period coincided with strengthening political commitment for policy and programming responses to advance breastfeeding internationally, and in Brazil, including significant advances in legal protections. This included the adoption of the NBCAL, and the inclusion in the Brazilian Constitution, of the right of all women to 120 days of maternity leave [30, 35].
3. **Mid-2000s onwards – breastfeeding rates plateau and CMF sales surge**: Between 2006 and 2013, growth in the prevalence of exclusive breastfeeding to six months plateaued, and then regressed slightly to 36.6%; and breastfeeding at one year to 45.4% [34, 36]. Despite the aforementioned policy responses and legal protections, CMF sales surged 750.0% between 2006 and 20, from R$278 million (US$62 million) to R$2,367 million (US$525 million) [37]. In 2020, the standard, follow-up and toddler milk categories generated R$838 million (US$ 186 million), R$512 million (US$114 million) and R$710 million (US$ 158 million) in sales respectively, growing between seven and eight-fold over the period. The specialized formula category grew 23-fold over the same period, from R$13.6 million to R$307 million [37].

### The baby food industry and the power of its marketing in Brazil

**Fig. 1:**
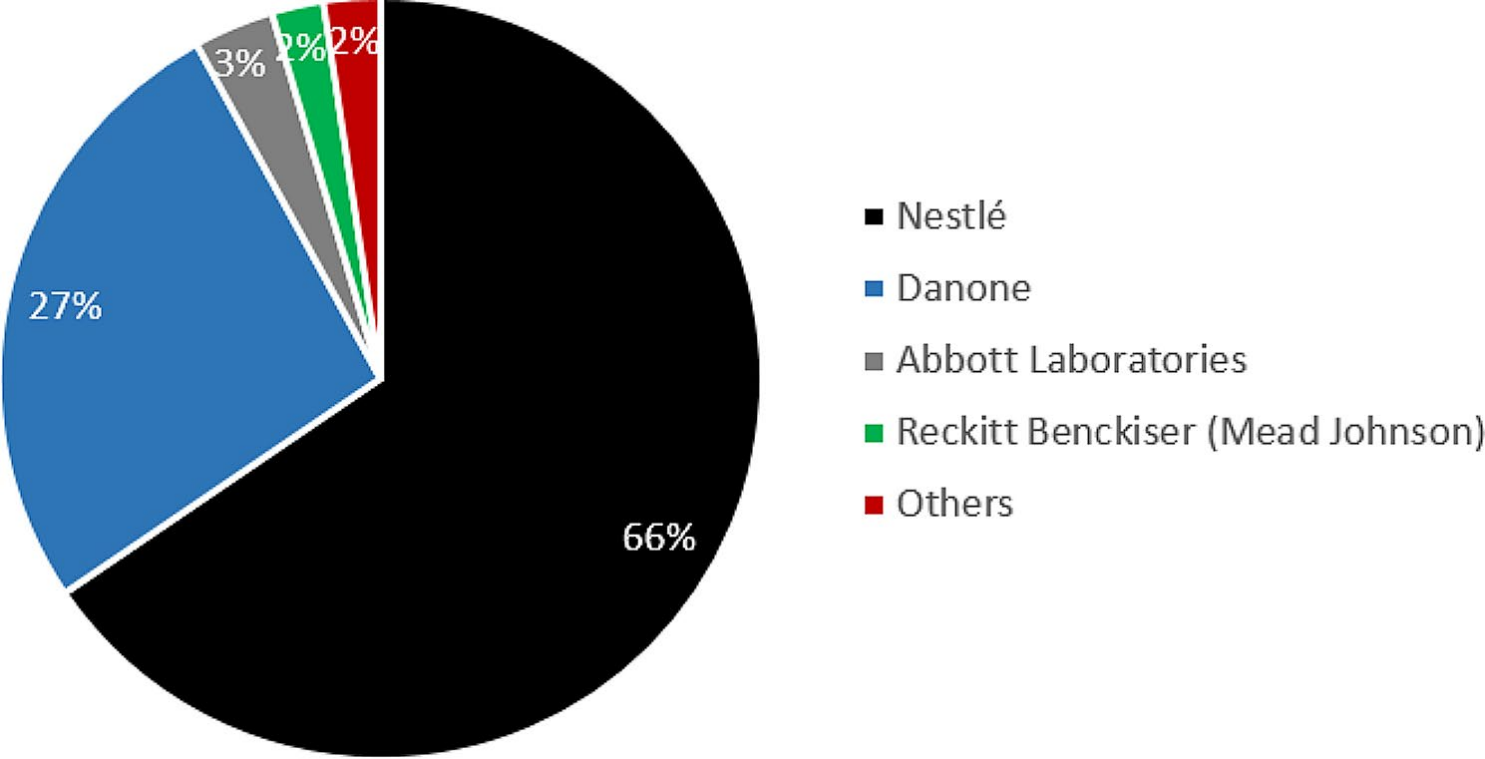
Brazil’s CMF market structure in 2020, showing % share of market leaders. Notes: Data sourced from Euromonitor Passport

The baby food industry has a long history in Brazil. Nestlé, the world’s largest food manufacturer headquartered in Switzerland, was at the vanguard of the industry’s first-wave of globalization in the late-1800s, hence benefiting from its ‘first-mover advantage’ in many markets [2]. This included Brazil, where the company established its first factory in 1921, producing condensed milk, and then by 1924, powdered milk products [38]. By 1946, the company had 14 sales offices nationwide, employing 669 people [39]. As of 2020, it was operating 16 factories across the country (producing all products, not just CMF) [40]. Danone has been present in the country since at least 1970, through its specialised nutrition division Nutricia [41]. Figure 1 shows that Nestlé is by far the market leader (65.5% market share), followed by Danone (France; 26.5%), Abbott Laboratories (United States; 3.4%) and Reckitt Benckiser Mead Johnson (United Kingdom; 2.2%). The industry is therefore strongly oligopolistic, with 92.0% market share accruing to just two transnational corporations.

Intensive marketing initiated by the industry coincided with the decline in breastfeeding and normalisation of CMF in Brazil [42], alongside the rising consumption of ultra-processed food products in the paediatric [43, 44] and wider population [45]. As in many other countries, standard marketing techniques were used, including direct-to-consumer advertising, health professional co-optation, and product strategies [8, 9]. Nestlé initiated its *Serviço de Colaboração Familiar* (Family Collaboration Service) in 1942, to connect directly with Brazilian families, alongside the creation of a new character, Ruth Beatriz, to advise mothers on how to feed their babies [46]. Wide-reaching mass-media campaigns promoting products were initiated in the 1960s [42].

In the mid-1980s, to expand their markets further and to avoid regulations they perceived as applying to infant formula only, the baby food industry began to heavily market follow-up formulas and toddler milks for older infants and young children, thereby ‘widening’ the age boundaries of their markets [2]. Follow-up formulas and toddler milks have been branded and labelled in nearly identical ways to infant formula, and thereby used to ‘cross-promote’ entire branded product ranges [8]. According to the sales data reported earlier in this paper, such products now comprise the majority of Brazil’s CMF sales [37]. In 2019, 13 different toddler milk brands were reportedly available to Brazilian consumers, heavily promoted on social media, and typically with various promotional claims on labels, alongside giveaways and price promotions [47].

### Corporate political activity of the baby food industry in Brazil

Since 1988, Brazil has had among the world’s strongest breastfeeding protection laws (see the following section) [18]. However, to sustain the power of its marketing, and to counter threats from the public health community, the industry has engaged in various CPA. We provide evidence of these activities below.

#### Building strategic alliances within the industry

First, in order to have a stronger voice in public policymaking and in wider public discourse, the companies have built strategic alliances within the industry. According to several informants and documentary evidence, the baby food industry is part of a wider set of powerful industry actors in the dairy and agricultural sectors, including for example:

*…a large conglomerate – [those] supplying milk, and the agribusiness sector…also has a super important role. It’s not just one or two companies* (I08).

Baby food corporations were also represented by industry lobby groups, such as the Brazilian Association of Food Industries for Special Purposes and Related (*Associação Brasileira da Indústria de Alimentos para Fins Especiais e Congêneres* – ABIAD, in Portuguese) which represents the dietary supplements industry, and the Brazilian Association of Food Industries (*Associação Brasileira da Indústria de Alimentos* – ABIA, in Portuguese) which is the national trade association for the food industry. These trade associations are a part of a larger global network of industry organizations, coordinated and funded by the companies [2, 6]. ABIAD is part of the Latin American Alliance for Responsible Nutrition (*Aliança Latino-Americana de Nutrição Responsável* – ALANUR, in Portuguese), an organization that claims to “promote scientific knowledge about nutritional supplements” [48].

However, ALANUR has direct ties with industry. The recent vice-president of ALANUR was a senior director for regulatory affairs at Glaxosmithkline (GSK) Brasil, a pharmaceutical company, and President of ABIAD [49]. Another person, the president of ALANUR works at Herbalife, a dietary supplement company, and is a member of the executive council of the International Life Sciences Institute (ILSI) Nor-Andino – the North Andean branch of ILSI [49]. ILSI is an international front group to the food industry, that has been shown to shape public policy and science across the globe in the interests of its corporate members [50, 51], and at the detriment of public health. Danone, Abbott and other companies are currently members of ILSI Nor-Andino [52].

#### Direct influence in public policymaking

The baby food industry and its lobbyists were described by our interviewees as having good access to policymakers and regulatory agencies in Brazil.

*They have several strategies, for example: inviting ministers to large events, (…) going to the Civil House [Executive Office of the Presidency of the Republic]. They are several [representatives from the industry] at the same time, traveling together with the Minister of Foreign Affairs - this is a strategy they use* (I06).

The Brazilian Health Surveillance Agency (*Agência Nacional de Vigilância Sanitária* – Anvisa, in Portuguese), has oversight for monitoring the *The Brazilian Code*. As part of Anvisa’s regular decision-making processes, the agency invites representatives of all interested organizations related to the subject, including industry. However, Anvisa was considered to be ‘permeated’ by industry lobbyists;

*Very present, for example, [is a] Mead Johnson representative [who] has entry without the need to identify themself (…) [the] industry enters and passes right in, clearly giving the impression that they enter at any time they want. (…) I witness it in person* (I01).

Several participants noted that this influence with Anvisa represents a major challenge for policy implementation. Indeed, neither Anvisa nor the baby food companies corporations were seen to react when civil society organizations notify them about violations of *The Brazilian Code*. Some participants perceived a weakness in the implementation of sanctions, when companies violated the law;

*We have a regulation that is the Brazilian Code, [and] its implementation depends a lot on the government. Surveillance…is led by Anvisa, but Anvisa is very permeated by the industry. In all governments, regardless of [the political party in power], the industry is always present* (I01).

Some interviewees nevertheless noted that it was less the individual companies themselves who participated in lobbying activities, but more the trade associations they have established, coordinate and fund;

*Whenever we are discussing a public policy – for example within the government or within the Chamber [of Deputies], or the Senate – it is not necessarily the industry itself that is talking. Not necessarily Nestlé, Mead Johnson or Danone. It is rather the Brazilian Association of Food Industries, ABIA* (I19).

There was a noted tension between the need for baby food corporations to combine forces in their lobbying, and in representing their own respective interests;

*We know that these industry associations are the same - the directors of ABIAD or ABIA are the ones who have put Nestlé forward to scrutinise those spaces. […] They say they are not speaking on behalf of [a given] company – that they are speaking from the group to discuss the health of the Brazilian child - but we know that this is not real* (I19).

Civil society was viewed by our participants as having more limited access to public policy decision makers within government relative to the industry. As one informant put it;

*[At Anvisa] I have to identify myself – [I] have difficulties engaging in dialogue [with the agency], but the industry comes and passes right in* (I01).

This was particularly so at the international level, with participants explaining that civil society organizations do not have the same level of participation in standard-setting spaces, such as the Codex Alimentarius Commission (Codex), at least partially due to limited economic and human resources. Codex is a common programme of WHO and the Food and Agriculture Organization of the United Nations (FAO), that sets standards related to food processing, labelling and safety, including CMF;

*[In 2019] Anvisa sent a communication to all members [of the Codex mailing list] asking who could make up the Brazilian delegation to go to the discussions. [Civil society organizations] do not have the resources to pay. So, it was Mead Johnson who was available for the trips to Berlin (Germany) [where Codex discussions took place]* (I01).

*In the forum that takes place at Anvisa, in the Ministry of Health, to deal with Codex, companies have a seat, they are not only here as representatives – but they are also represented as an industry, as they are also represented by the Association of the Industries* (I16).

#### Capturing Brazil’s pediatric profession

The baby food industry has also taken action to influence the infant and young child feeding knowledge environment in Brazil, including through its co-optation of major health professional associations, and activities to shape professionals norms and practices. Medical endorsement by professionals helps baby food corporations to bolster their legitimacy with the public and policymakers, and to promote their products as safe, scientific and medically endorsed [6].

Nestlé has a long-standing relationship with the Brazilian Society of Pediatrics (*Sociedade Brasileira de Pediatria* – SBP, in Portuguese), and other regional paediatric associations [53]. Several scientific and technical documents developed by the SBP are sponsored by Nestlé [54, 55]. As of 2020, a Nestlé representative was a member of the SBP’s Executive Board, and the Board of Trustees of the SBP’s Foundation. Nestlé has also run a training course in collaboration with the SBP since 1956, the *Curso Nestlé de Atualização em Pediatria* (Nestlé Update Course in Pediatrics, in English), with its 69th edition attended by more than “five thousand professionals” in 2020 [38, 56].

In June 2020, Nestlé and SBP launched a paediatric residents training program on infant nutrition, called the *Programa Jovens Pediatras* (Young Paediatricians Programme) [57]. The opening ceremony for the programme was attended by representatives from Nestlé, as well as a paediatrician who coordinated a programme funded by Danone, called ‘O Nutri-Brasil Infância’ (“Nutri-Brasil Childhood”, in English) [58]. Nestlé Nutrition was a sponsor of the ‘Walk for the Valuation of Pediatricians’ organized by SBP and carrying the slogan ‘Who goes to the Pediatrician comes back Quiet’, with support for the campaign from a prominent Brazilian actress [59]. ‘Danone also promoted a “Gastro Virtual Conference”, targeted at paediatric gastroenterologists and delivered in partnership with the SBP in 2020 [53]. In 2020, the Brazilian Society of Nutrology (*Sociedade Brasileira de Nutrologia* – SBN, in Portuguese) received financial support from the Danone Nutricia to make the “Consensus of the Brazilian Association of Milk Nutrition for Children from 1 to 5 years old” viable [60].

Several participants further noted that health professionals are sponsored by baby food corporations to attend scientific events, for example;

*SBP holds a Brazilian Congress of Paediatrics in different cities around the country every 2 to 3 years. In this congress, [baby food companies] invite[s] the speakers and pays for everything (…) they pay for dinners, the hotel, someone who will pick them up at the airport* (I01).

SBP continued to work in partnership with the baby food industry during the COVID-19 pandemic, with different events sponsored by Danone on social media, with a focus, for example, on “difficulty in childhood growth: a practical guide for nutritional diagnosis and management” [61], and “telemedicine in times of the new coronavirus” [62]. ILSI Brasil, mentioned earlier, had a nutrition taskforce on child nutrition, led by baby food corporations, and was found to have organised a round table in an international conference on child health in 2018 [58]. As found extensively in the United Kingdom [63, 64], the baby food industry also has close connections with allergists. Six of the 13 paediatricians who authored a study on the adherence of paediatricians in Brazil to food allergy guidelines declared conflicts of interest because of their associations with Danone and/or Nestlé [65].

### Civil society mobilization, political commitment and policy responses

In this section we present evidence of actions by public health actors and coalitions in Brazil to generate political commitment for a strong national policy framework for the protection, promotion and support of breastfeeding and public health. The purpose of this section is to demonstrate that the power of the baby food industry in Brazil’s first food system has not been unchallenged; rather, policy and regulatory successes have resulted from civil society mobilization, and actions by the government to resist and counteract the industry’s marketing and political activities. In the words of one informant;

*Brazil has a very beautiful trajectory in the promotion of breastfeeding - there is a lot of strength from an organized civil society, academia, health professionals, the Ministry of Health - there is always a lot of fight* (I06).

In the 1970s, reports by health professionals and civil society groups, including the report *The Baby Killer* published by the NGO War on Want, revealed how the aggressive marketing and promotion of BMS was driving a worldwide decline in breastfeeding, and the use of formula was contributing to the deaths of infants in multiple countries [66]. This triggered worldwide public scrutiny, the birth of the International Baby Food Action Network (IBFAN) and many member organizations at the country-level, and what was to become the largest international boycott against a food company in history, against Nestlé as the market leader at the time [67, 68]. In direct response to these developments, WHO and UNICEF initiated the development of the International Code of Marketing of Breast-milk Substitutes, adopted by WHO member states, including Brazil, in 1981 [2, 14, 67].

Momentum to protect, promote and support breastfeeding was already building in Brazil. In 1976, the government adopted the National Food and Nutrition Program (*Programa Nacional de Alimentação e Nutrição* – PRONAN, in Portuguese), and then in 1981 in response to growing civil society advocacy, the comprehensive National Breastfeeding Incentive Program (*Programa Nacional de Incentivo ao Aleitamento Materno* – PNIAM, in Portuguese) [69]. Both were recognised as key policy pillars that enabled the country’s breastfeeding resurgence [35, 70]. In 1983, IBFAN-Brazil was founded, and by 2004 it had 72 member branches operating across 18 states and 39 cities [71]. In 1988, *The Code* was implemented into national law, as the NBCAL (*The Brazilian Code*) [72]. Oversight for monitoring was assigned to Anvisa and it’s surveillance system (*Sistema Nacional de Vigilância Sanitária* – SNVS, in Portuguese), comprising local health surveillance units (*Vigilâncias Sanitárias* – VISAs, in Portuguese) coordinated by Anvisa [73].

In 1988, the right to health was enshrined in the National Constitution, alongside the establishment of the Unified Health System (*Sistema Único de Saúde* – SUS, in Portuguese), and the right to maternity leave, and of women’s freedom to remain with their baby during breastfeeding [74]. Further programmes were established, including the development of a national breastmilk bank network between 1985 and 1988. In 1990, the government affirmed its commitment to promote, protect and support breastfeeding at the Children’s Summit in New York [75], followed by the adoption of the Statute of the Child and Adolescence (*Estatuto da Criança e do Adolescente* – ECA, in Portuguese) in 1990. This was further bolstered by the introduction of programs, including the WHO/UNICEF Baby Friendly Hospitals Initiative, and the national network of human milk banks [11].

In response to evolving evidence and guidance, *The Brazilian Code* was strengthened in 1992, 2001, and 2002, to cover not only infant formulas and bottles, but also pacifiers, toddler milks and commercial complementary foods [73, 76, 77]. Following the election of the leftist Lula administration in 2003, the government implemented major social policy reforms, including the Fome Zero (Zero Hunger, in English) and the Bolsa Familia (Family Allowance, in English) welfare programmes, which contributed to significant reductions in malnutrition and poverty, including rapid reductions in child stunting. In 2010, the right to food was explicitly recognised in the Brazilian Constitution, by Congressional amendment.

In 2013, the Breastfeeding and Feeding Brazil Strategy (*Estratégia* Amamenta e Alimenta Brasil – EAAB, in Portugese) was adopted, involving actions to promote, protect and support breastfeeding and healthy complementary feeding for children under two, improving the skills and abilities of health professionals in primary health care (BRASIL, 2013). This was seen as generating significant momentum, with multiple actors convened to inform the strategy;

*We had for a long time in the National Breastfeeding Committee [who had] links with the Ministry of Health, where [the public health community] participated in this policy discussion (…), also the professionals: nursing, obstetrics and gynaecology associations - FEBRASGO, the Brazilian Society of Paediatrics, IBFAN (…), representatives from women’s groups* (I09).

In 2021, another important development was the publication of the Dietary Guidelines for Brazilian Children Under Two Years of Age (*Guia Alimentar para Crianças Brasileiras Menores de 2 Anos*, in Portuguese), published by the Ministry of Health [78]. This new edition aligns with the Dietary Guidelines for the Brazilian Population (*Guia Alimentar para a População Brasileira*, in Portuguese) (2014), by adopting the Nova classification as a reference and contributing to the promotion of healthy eating patterns and the protection of food cultures, including the recommendation that ultra-processed foods should not be given to children.

The civil society organizations IBFAN-Brasil, and the Brazilian Institute for Consumer Defense (*Instituto Brasileiro de Defesa do Consumidor* – Idec, in Portuguese), have played crucial social accountability roles by monitoring the implementation of the NBCAL and its violations by baby food corporations, which have been reported on annually [79, 80]. IBFAN-Brasil was central in convening the 3rd World Conference on Breastfeeding in Rio de Janeiro in 2019, which generated significant visibility for breastfeeding amongst government officials and the wider public, including through a women’s mass breastfeeding event. Furthermore, IBFAN-Brazil has provided regular ongoing training of *‘olhos vivos’* (living eyes), including health inspectors, to monitor and report on those violations.

In 2016, the civil society Alliance for Adequate and Healthy Diets (*Aliança pela Alimetnação Adequada e Saudável*, in Portuguese) was launched, comprising multiple public health, nutrition, food security and environmental organizations [81]. The Alliance focuses its advocacy on 10 key themes, including one on breastfeeding and health complementary feeding.

## Discussion

In this study, our aim was to describe and understand the power of the baby food industry, and in particular the market and political strategies it has used to shape Brazil’s first-food system, to drive and sustain infant and young child diets high in CMF. We further investigated how breastfeeding advocates and coalitions, and policymakers, have resisted this influence to develop a strong national breastfeeding protection law and policy framework.

### The power of mik formula marketing in Brazil

Similar to an earlier case study in the Philippines [3], intensive marketing initiated by the baby food industry coincided with declining breastfeeding rates and the normalisation of CMF in Brazil during the mid and late-20th Century, alongside the rising consumption of many other ultra-processed food products in the country [45]. Just as ultra-processed foods have displaced traditional diets, comprising minimally processed meals and cuisines, CMF have displaced breastfeeding as a bio-psycho-social system of nutrition and care. The marketing practices we report in Brazil, are highly consistent with the strategies reported in other countries and worldwide [8, 10, 13, 78].

The sales data we report in this study show Brazil’s CMF market grew 7.5-fold, in just 14 years between 2006 and 2020. As reported elsewhere, the industry has managed to grow sales markedly in the follow-up formula, toddler milks and specialized formula categories. Rapid growth in follow-up formula and toddler milks, which together now comprise more than half the industry’s total sales in Brazil, is remarkable, given WHO has long considered these products to be unnecessary for a healthy infant and young child diet, and unsuitable as breastmilk substitutes [82]. Such products are typically much more expensive than regular animal milks or milk substitutes, thereby unnecessarily diverting household expenditure from basic necessities, such as nutritious food, health care and education [6].

The rapid reported growth in specialised milks deserves much more scrutiny, given evidence from other countries of industry-driven over-diagnosis of cows milk protein allergy [63, 64], as well as evidence the industry is pathologizing normal infant behaviours such as sleeplessness, to sell products claiming to deliver treatments [8, 83]. We found no studies on the marketing of CMF through social and other digital media in Brazil. This represents a major gap in research, especially given evidence of the wide scope and impact of such marketing elsewhere, and given recent attention to this issue by WHO member states [84].

### The power to market milk formulas in Brazil

Our findings also show that, in order to support and sustain the power of its marketing in Brazil, the baby food industry has implemented political strategies to influence infant and young child feeding policy, regulation and knowledge environment.

We find the industry has used front groups to engage in its lobbying practices, including not only those concerned with infant nutrition, but also with agri-business and the wider food industry, and in disseminating corporate-backed science. By outsourcing their lobbying to these front groups the corporations can distance themselves from reputational damage, while promoting an image of corporate social responsibility, including their stated support for breastfeeding [2, 6]. These industry front groups we report in Brazil, are part of a much wider international network of similar groups, which are established and coordinatd by the companies. Lobbying by this international network is recognsied as a major barrier to strengthening national breastfeedign protection laws worldwide, and to implementation of *The Code* [2]. We found that lobbyists working on behalf of the baby food industry in Brazil likely had direct access to government officials, indicating the potential to influence policy-makers beliefs and practices, and ultimately the potential to influence (or block) legislative change. In contrast, we found indications that civil society groups had much more limited access.

Similar to findings of a recent study in France [85], we also report how the baby food industry has co-opted major health professional associations, to influence professional norms and practices. This includes not only through it’s marketing activities we described earlier, but also through deep and long-standing linkages between baby food corporations, and especially Nestlé and Danone, and pediatric associations. These relationships help companies to portray themselves as experts on infant and young child nutrition, and to enhance their legitimacy with health professionals, policy-makers and civil society [85]. Such linkages create conflicts of interest, and ultimately compromise the integrity of health professionals providing infant and young child nutrition and maternity care in Brazil [8, 86], and call into question the policies and practices of the Sociedade Brasileira de Pediatria (SBP) as the leading pediatric association in the country. The SBP and leaders within the pediatric profession, should act to urgently addresss this conflicting interest, and show leadership by ending all relationships with the baby food industry, as others have done and called for elsewhere [8, 87, 88].

We also report how, despite the activities of the baby food industry described above, Brazil has managed to develop a strong national breastfeeding protection law and policy framework, and has developed world-leading innovations in dietary guidelines for infants and young children. The mobilization of civil society groups in the country, connected with wider movements for breastfeeding, appears to have been vitally important. This has occurred alongside periods of stronger political commitment for breastfeeding in the country, and constitutional amendments and social policy reforms that enshrine the right to health, food and breastfeeding. Despite Brazil’s strong national breastfeeding protection law, in the form of *The Brazilian Code*, sales of milk formula products have rapidly escalated in the country. This suggests that government, working with civil society groups and experts, should take action to strengthen policy even further, to curb newer powerful forms of marketing, including digital marketing described earlier.

### Strengths and limitations of this study

Our study has limitations. First, although we report evidence that industry lobbyists have good access to government policymakers, we do not provide evidence of what their interactions involve substantively, or how these interactions have impact on policy development or implementation. We found access to publically available documentation on industry’s lobbying activities very limited, due to lack of transparency and reporting on lobbying in Brazil more generally.

Second, due to this limited data availability, we believe our reporting of the industry’s political activities is part of a major wider system of corporate influence in the country. Third, no industry representatives agreed to be interviewed, and so we could not get their perspective of these issues. Many of the actions presented in our manuscript do not happen in public spaces. Ethnographic studies, with the immersion of researchers in communities, for example, might be useful to better identify CPA practices used by the baby food indsutry in different contexts.

Fourth, the use of trade associations by the baby food industry in Brazil for its representation in public policy has proved to be a challenge for our study, since those trade associations do not necessarily speak overtly on behalf of companies and cover many other sectors than infant formula (such as dietary supplements, non-alcoholic beverages etc.). Nevertheless, our interviews were crucial in revealing the extent of the practices used by the industry in Brazil. Fifth, we undertook the study during the early stages of the covid-19 pandemic, and we may have had more interviewees under different conditions.

Sixth, some CPA described in our manuscript may no longer be used, and regular monitoring of these practices is needed to see if they evolve over time, or with different political parties in power. Some interviewees noted, for example, that some commissions in government in charge of infant and young child nutrition were disrupted after the Bolsonaro administration took power in 2018. This may have negatively impacted breastfeeding, or been beneficial to the baby food industry, if breastfeeding protection laws or associated monitoring and enforcement systems were weakened, or if new public health initiatives were not prioritized or implemented.

Seventh, we have focused on the power of the industry’s marketing, but have not elaborated on the power of specific health promotion and social marketing iniatives or campaigns intended to promote and support breastfeeding. Our focus has instead focused largely on policy and political level developments, which could be perceived as ‘top-down’ in orientation. However, we acknowledge the importance of combining a ‘top-down’ and ‘bottom-up’ approach, which is supported by evidence of what works to improve breastfeeding and public health at the population level [83]. Furthermore, we recognize that industry marketing can also be conceptualized as operating across these multiple levels, targeting not just consumers, but also professionals, policy-makers and politicians [8, 89]. 

Finally, we have not reported on levels of financing for breastfeeding, even though this is an important indicator of political commitment for the implementation of the country’s breastfeeding policy framework. We have also not considered the rise of commercial complementary foods in the country, although these foods are now a significant share of children’s diets. These are topics for future research.

## Conclusion

We find that the baby food industry has powerfully shaped Brazil’s first-food system in ways that promote and sustain infant and young child diets high in CMF, to the detriment of breastfeeding. This industry uses a number of political strategies to protect and sustain its CMF marketing, through actions targeting policymakers and health professionals especially. Overall, our findings reveal that corporate political activity by this industry is likely to be an important barrier to strengthening future actions to protect, promote and support breastfeeding women and families in Brazil. This suggests that new modalities of public health action are needed to reudce corporate power over Brazil’s first-food system, including actions to limit the industry’s access to policymakers in the country, while ensuring that civil society groups and professionals without conflicting interests are engaged to guide decision-making and ensure accountability.

Priority action should also include the elimination of conflicts of interet within the pediatric profession in Brazil, which ultimately undermines the credibility of the profession and calls into question the impartiality of the guidance it provides to health professionals, and the advice that pediatricians given to women and families. At a miminimum this could include ending financial relationships that professional associations have with the industry, preventing and managing conflicts of interest among members, and preventing participation by industry in professionl training and conferences.

Ultimately, such actions will help to advance the rights to health, food and nutrition of the Brazilian people, and most of all the rights of mothers and children, over vested commercial interests.

### Electronic supplementary material

Below is the link to the electronic supplementary material.

Supplementary Material 1

## Data Availability

All data generated or analysed during this study are included or cited in this published article. The information contained in this manuscript has been obtained from sources believed to be reliable. However, any potential interpretation of the findings as making an allegation against a specific named company, companies or persons would be incorrect and misleading.

## References

[1] Gilmore AB, Fabbri A, Baum F, Bertscher A, Bondy K, Chang H-J et al. Defining and conceptualising the commercial determinants of health. The Lancet. 2023. 10.1016/S0140-6736(23)00013-2 36966782

[2] Baker P, Russ K, Kang M, Santos TM, Neves PA, Smith J (2021). Globalization, first-foods systems transformations and corporate power: a synthesis of literature and data on the market and political practices of the transnational baby food industry. Globalization and Health.

[3] Baker P, Zambrano P, Mathisen R, Singh-Vergeire MR, Escober AE, Mialon M (2021). Breastfeeding, first-food systems and corporate power: a case study on the market and political practices of the transnational baby food industry and public health resistance in the Philippines. Globalization and Health.

[4] Cossez E, Baker P, Mialon M. ‘The second mother’: how the baby food industry captures science, health professions and civil society in France. Matern Child Nutr. 2021:e13301. 10.1111/mcn.13301 PMC8932685 34935291

[5] Tanrikulu H, Neri D, Robertson A, Mialon M (2020). Corporate political activity of the baby food industry: the example of Nestlé in the United States of America. Int Breastfeed J.

[6] Baker P, Smith JP, Garde A, Grummer-Strawn LM, Wood B, Sen G et al. The political economy of infant and young child feeding: confronting corporate power, overcoming structural barriers, and accelerating progress. The Lancet. 2023. 10.1016/S0140-6736(22)01933-X 36764315

[7] World Health Organization. Global Strategy for Infant and Young Child Feeding. Geneva; 2003.

[8] Rollins N, Piwoz E, Baker P, Kingston G, Mabaso KM, McCoy D (2023). Marketing of commercial milk formula: a system to capture parents, communities, science, and policy. The Lancet.

[9] World Health Organization. How the marketing of formula milk influences our decisions on infant feeding. Geneva. ; 2022. Available from: https://www.who.int/publications/i/item/9789240044609.

[10] Piwoz EG, Huffman SL (2015). The impact of marketing of breast-milk substitutes on WHO-recommended breastfeeding practices. FoodNutr Bull.

[11] Rollins NC, Bhandari N, Hajeebhoy N, Horton S, Lutter CK, Martines JC (2016). Why invest, and what it will take to improve breastfeeding practices?. The Lancet.

[12] Hastings G, Angus K, Eadie D, Hunt K (2020). Selling second best: how infant formula marketing works. Globalization and Health.

[13] Baker P, Santos T, Neves PA, Machado P, Smith J, Piwoz E, et al. First-food systems transformations and the ultra-processing of infant and young child diets: the determinants, dynamics and consequences of the global rise in commercial milk formula consumption. Matern Child Nutr. 2021;17(2):e13097. 10.1111/mcn.13097 PMC7988871 33145965

[14] Richter J (2001). Holding corporations accountable: corporate conduct, international codes, and citizen action.

[15] World Health Organization. Marketing of Breast-Milk Substitutes: National Implementation of the International Code Status Report. Geneva. ; 2020. Available from: https://www.who.int/publications/i/item/9789240006010.

[16] Granheim SI, Engelhardt K, Rundall P, Bialous S, Iellamo A, Margetts B (2017). Interference in public health policy: examples of how the baby food industry uses tobacco industry tactics. World Nutr.

[17] Cetthakrikul N, Baker P, Banwell C, Kelly M, Smith J (2021). Corporate political activity of baby food companies in Thailand. Int Breastfeed J.

[18] World Health Organization. Marketing of breast-milk substitutes: national implementation of the International Code, Status report 2022. Geneva. ; 2022. Available from: https://iris.who.int/bitstream/handle/10665/354221/9789240048799-eng.pdf?sequence=1.

[19] Yin R (2017). Case study research and applications: design and methods.

[20] George AL, Bennett A (2005). Case studies and theory development in the social sciences.

[21] Kay A, Baker P (2015). What can causal process tracing offer to policy studies? A review of the literature. Policy Stud J.

[22] The World Bank. World Development Indicators, Washington DC. ; 2022. Available from: https://databank.worldbank.org/source/world-development-indicators.

[23] Federal Government of the United States., editor. The World Factbook - Brazil. Washington, DC: Central Intelligence Agency.

[24] Lee K, Chagas LC, Novotny TE (2010). Brazil and the framework convention on tobacco control: global health diplomacy as soft power. PLoS Med.

[25] Kickbusch I, Silberschmidt G, Buss P (2007). Global health diplomacy: the need for new perspectives, strategic approaches and skills in global health. Bull World Health Organ.

[26] United Nations Human Rights Office of the High Commissioner. Ratification Status for Philippines. Geneva. ; 2022. Available from: https://tbinternet.ohchr.org/_layouts/15/TreatyBodyExternal/Treaty.aspx?CountryID=24&Lang=EN.

[27] Mialon M, Swinburn B, Sacks G (2015). A proposed approach to systematically identify and monitor the corporate political activity of the food industry with respect to public health using publicly available information. Obes Rev.

[28] Goodman LA. Snowball sampling. The annals of mathematical statistics. 1961:148–70.

[29] Sousa PLR (1975). The decline of breastfeeding in Brazil. J Trop Pedat Env Chld Hlth.

[30] Venancio SI, Saldiva SRDM, Monteiro CA (2013). Secular trends in breastfeeding in Brazil. Rev Saúde Pública.

[31] Buccini GS, Pérez-Escamilla R, Venancio SI (2016). Pacifier use and exclusive breastfeeding in Brazil. J Hum Lactation.

[32] Buccini G, Pérez-Escamilla R, D’Aquino Benicio MH, Justo Giugliani ER, Isoyama Venancio S (2018). Exclusive breastfeeding changes in Brazil attributable to pacifier use. PLoS ONE.

[33] Victora CG, Aquino EM, do Carmo Leal M, Monteiro CA, Barros FC, Szwarcwald CL (2011). Maternal and child health in Brazil: progress and challenges. The Lancet.

[34] Boccolini CS, Boccolini PMM, Monteiro FR, Venâncio SI, Giugliani ERJ. Breastfeeding indicators trends in Brazil for three decades. Rev Saúde Pública. 2017; 51. 10.11606/S1518-8787.2017051000029 PMC5697916 29166437

[35] Melo DS, Oliveira, MHd, Pereira DdS. Brazil’s progress in protecting, promoting and supporting breastfeeding from the perspective of the global breastfeeding collective. Revista Paulista De Pediatria. 2020; 39. 10.1590/1984-0462/2021/39/2019296 PMC7457464 32876303

[36] Wagner KJP, de Fragas Hinnig P, Rossi CE, de Almeida Alves M, Leite MS (2020). De Assis Guedes De Vasconcelos F. Time trends in the prevalence of breastfeeding among schoolchildren from public and private schools in Florianópolis, Southern Brazil: from 2002 to 2013. Am J Hum Biology.

[37] Euromonitor International. Passport. London. ; 2022. Available from: https://www.euromonitor.com/our-expertise/passport.

[38] Nestlé Brasil. History. ; 2021. Available from: https://www.Nestlé.com.br/a-Nestlé/historia.

[39] Fazwal A, Holla R. The Boycott Book. online; Self-published ; 2019. Available from: http://www.theboycottbook.com/intro.pdf.

[40] Nestle SA, Annual Review. 2019. Vevey; 2019. Available from: https://www.nestle.com/sites/default/files/2020-03/2019-annual-review-en.pdf.

[41] Danone Nutricia. Who we are. ; 2021. Available from: https://www.danonenutricia.com.br/quem-somos.

[42] Oliveira DSd, Boccolini CS, Faerstein E, Verly-Jr E (2017). Breastfeeding duration and associated factors between 1960 and 2000. Jornal De Pediatria.

[43] Bielemann RM, Santos LP, dos Santos Costa C, Matijasevich A, Santos IS (2018). Early feeding practices and consumption of ultraprocessed foods at 6 y of age: findings from the 2004 Pelotas (Brazil) Birth Cohort Study. Nutrition.

[44] Dallazen C, Silva SAd, Gonçalves VSS, Nilson EAF, Crispim SP, Lang RMF et al. Introduction of inappropriate complementary feeding in the first year of life and associated factors in children with low socioeconomic status. Cadernos De Saude Publica. 2018; 34. 10.1590/0102-311X00202816 29489953

[45] Monteiro CA, Levy RB, Claro RM, de Castro IRR, Cannon G (2010). Increasing consumption of ultra-processed foods and likely impact on human health: evidence from Brazil. Public Health Nutr.

[46] Coelho L, Peres J. How Nestlé appropriated Brazilian recipes (or how we became the country of condensed milk); o jojo e o trigo; 2021. Available from: https://ojoioeotrigo.com.br/2021/04/como-a-nestle-se-apropriou-das-receitas-brasileiras-ou-de-como-viramos-o-pais-do-leite-condensado/.

[47] Oliveira Dias Leão D, Bauermann Gubert M. Precisamos conversar sobre os chamados compostos lácteos (we need to talk about so-called growing-up milks). Volume 14. Alimentação, Nutrição & Saúde; 2019. 0.

[48] Alianza Latinoamericana de Nutricion Responsable, About ALANUR. ; Chicago2023. Available from: https://alanurla.org/en/about-alanur/.

[49] LinkedIn Nwithheld. ; 2023. Available from: https://www.linkedin.com/in/jos%C3%A9-orteg%C3%B3n-408635133/.

[50] Steele S, Ruskin G, Sarcevic L, McKee M, Stuckler D (2019). Are industry-funded charities promoting advocacy-led studies or evidence-based science? A case study of the international Life Sciences Institute. Globalization and Health.

[51] Mialon M, Ho M, Carriedo A, Ruskins G, Crosbie E (2021). Food industry shaping of the principles of scientific integrity. Eur J Pub Health.

[52] Andean IN. Industry members; Bogotá2023. Available from: https://ilsinorandino.org/miembros/.

[53] Melo M, Iwasawa N. Childhood in the crosshairs: Nestlé ‘attacks’ nutritionists and pediatricians in the pandemic; o jojo e o trigo; 2020. Available from: https://ojoioeotrigo.com.br/2020/09/infancia-na-mira-nestle-aponta-para-nutricionistas-e-pediatras-na-pandemia/.

[54] Sociedade Brasileira de Pediatria (SBP), Departamento Científico de Nutrologia. Nutrologia Pediátrica: Temas da Atualidade em Nutrologia Pediátrica– 2021. São Paulo.; 2021. Available from: https://www.sbp.com.br/fileadmin/user_upload/Manual_de_atualidades_em_Nutrologia_2021_-_SBP_SITE.pdf.

[55] Sociedade Brasileira de Pediatria (SBP)., Departamento Científico de Nutrologia. Manual de Suporte Nutricional da Sociedade Brasileira de Pediatria. 2020 [Accessed: 14 Feb 2023]. Available from: https://www.sbp.com.br/fileadmin/user_upload/2a_Edicao_-_jan2021-Manual_Suporte_Nutricional_-.pdf.

[56] Nestlé Brasil. Curso Nestlé de Atualização em Pediatria começa amanhã, no Rio de Janeiro.; 2012. Available from: https://www.nestle.com.br/media/pressreleases/cursonestledeatualizacaoempediatriacome%C3%A7aamanha%2Cnoriodejaneiro.

[57] Sociedade Brasileira de Pediatria. SBP e Nestle lancam programa para capacitar residentes de pediatria em temas de nutricao infantil.; 2020. Available from: https://www.sbp.com.br/imprensa/detalhe/nid/sbp-e-nestle-lancam-programa-para-capacitar-residentes-de-pediatria-em-temas-de-nutricao-infantil/.

[58] Mialon M, Cediel G, Jaime PC, Scagliusi FB (2022). A consistent stakeholder management process can guarantee the ‘social license to operate’: mapping the political strategies of the food industry in Brazil. Cadernos De saúde pública.

[59] Sociedade de Pediatria do Rio Grande do Sul. Caminhada Pela Valorização do Pediatra, em Canela. Journal SPRS; 2010.

[60] Nogueira-de-Almeida CA, Falcão MC, Ribas-Filho D, Zorzo RA, Konstantyner T, Ricci R (2020). Consensus of the Brazilian Association of Nutrology on milk feeding of children aged 1–5 years old. Int J Nutrology.

[61] Sociedade Brasileira de Pediatria. Guia pratico sobre dificuldade de crescimento na infancia e lancado durante live realizada pela SBP.; 2020. Available from: https://www.sbp.com.br/imprensa/detalhe/nid/guia-pratico-sobre-dificuldade-de-crescimento-na-infancia-e-lancado-durante-live-realizada-pela-sbp/.

[62] Sociedade Brasileira de Pediatria. Telemedicina e covid-19 SBP promovera live para orientar os pediatras acerca do tema.; 2020. Available from: https://www.sbp.com.br/imprensa/detalhe/nid/telemedicina-e-covid-19-sbp-promovera-live-para-orientar-os-pediatras-acerca-do-tema/.

[63] Munblit D, Perkin MR, Palmer DJ, Allen KJ, Boyle RJ (2020). Assessment of evidence about common infant symptoms and cow’s milk allergy. JAMA Pediatr.

[64] van Tulleken C. Overdiagnosis and industry influence: how cow’s milk protein allergy is extending the reach of infant formula manufacturers. BMJ. 2018; 363.

[65] Vieira SCF, Santos VS, Franco JM, Nascimento-Filho HM, Barbosa KdOeSS L-J, DPd (2020). Brazilian pediatricians’ adherence to food allergy guidelines—A cross-sectional study. PLoS ONE.

[66] Muller M. The baby killer: a War on want investigation into the promotion and sale of powdered baby milks in the Third World. London: War on Want; 1979.

[67] Sikkink K (1986). Codes of conduct for transnational corporations: the case of the WHO/UNICEF code. Int Org.

[68] Sokol EJ. The code handbook: a guide to implementing the international code of marketing of breastmilk substitutes. The code handbook: a guide to implementing the International Code of Marketing of Breastmilk Substitutes2013. p. 395-.

[69] Araújo M, Del Fiaco A, Werner EH, Schmitz B (2003). Incentivo Ao aleitamento materno no Brasil: Evolução do Projeto Carteiro Amigo Da Amamentação De 1996 a 2002. Revista Brasileira De Saúde Materno Infantil.

[70] Rea MF (1990). The Brazilian national breastfeeding program: a success story. Int J Gynecol Obstet.

[71] Allain A (2005). Fighting an old battle in a new world: how IBFAN monitors the baby food market.

[72] Bertoldo LAA, Oliveira MICd, Boccolini CS. Violations in the marketing of milks and complementary foods that compete with breastfeeding in Rio De Janeiro City, Brazil. Revista Paulista De Pediatria. 2022; 41. 10.1590/1984-0462/2023/41/2021228 PMC9273118 35830162

[73] Prado ISCF, Rinaldi AEM (2020). Compliance of infant formula promotion on websites of Brazilian manufacturers and drugstores. Rev Saude Publica.

[74] Melo D, Venancio S, Buccini G (2022). Brazilian strategy for Breastfeeding and complementary feeding Promotion: a Program Impact Pathway Analysis. Int J Environ Res Public Health.

[75] Sokol EJ (2005). The code handbook: a guide to implementing the international code of marketing of Breastmilk substitutes.

[76] Ministério da Saúde (BR)., Agência Nacional de Vigilância Sanitária DCA. Resolução RDC Nº 221, de 5 de agosto de 2002. Aprova o Regulamento Técnico sobre Chupetas, Bicos, Mamadeiras e Protetores de Mamilo. Brasília; 2022 [cited 17 Dec 2022]. Available from: http://ibfan.org.br/legislacao/pdf/rdc221.pdf.

[77] Ministério da Saúde (BR), Agência Nacional de Vigilância Sanitária DCA. Resolução da Diretoria Colegiada (RDC) nº 221, de 5 de agosto de 2002. Aprova o Regulamento Técnico sobre Chupetas, Bicos, Mamadeiras e Protetores de Mamilo. Brasília. ; 2022 [cited 17 Dec 2022]. Available from: https://bvsms.saude.gov.br/bvs/saudelegis/anvisa/2002/res0221_05_08_2002.html.

[78] Ministry of Health of Brazil, Secretariat of Primary Health Care, Health Promotion Department. Dietary guidelines for brazilian children under 2 years of age. Brasília. ; 2021. Available from: https://bvsms.saude.gov.br/bvs/publicacoes/dietary_guidelines_brazilian_chhildren_under.pdf.

[79] Instituto Brasileiro de Defesa do Consumidor (IDEC). Em defesa da amamentação e da alimentação complementar saudável; 2020 [cited 2023 01 Mar]. Available from: https://idec.org.br/defesa-da-amamentacao/monitoramento.

[80] Brasil IBFAN. NBCAL 2020 monitoring; 2020. Available from: http://www.ibfan.org.br/site/monitoramento-da-nbcal-2020.

[81] Alliance for Adequate and Healthy Diets. Alliance for Adequate and Healthy Food. ; 2020. Available from: https://alimentacaosaudavel.org.br/.

[82] World Health Organization. Information concerning the use and marketing of follow-up formula. Geneva. ; 2013. Available from: http://www.who.int/nutrition/topics/WHO_brief_fufandcode_post_17July.pdf.

[83] Pérez-Escamilla R, Tomori C, Hernández-Cordero S, Baker P, Barros AJ, Bégin F (2023). Breastfeeding: crucially important, but increasingly challenged in a market-driven world. The Lancet.

[84] World Health Organization. Scope and impact of digital marketing strategies for promoting breastmilk substitutes. Geneva. ; 2022. Available from: https://apps.who.int/iris/bitstream/handle/10665/353604/9789240046085-eng.pdf?sequence=2.

[85] Cossez E, Baker P, Mialon M (2022). The second mother’: how the baby food industry captures science, health professions and civil society in France. Matern Child Nutr.

[86] Grummer-Strawn LM, Holliday F, Jungo KT, Rollins N (2019). Sponsorship of national and regional professional paediatrics associations by companies that make breast-milk substitutes: evidence from a review of official websites. BMJ open.

[87] Mayor S (2019). Royal college stops taking funding from formula milk firms. BMJ.

[88] Godlee F, Cook S, Coombes R, El-Omar E, Brown N (2019). Calling time on formula milk adverts. BMJ.

[89] Bhattacharya CB, Korschun D (2008). Stakeholder marketing: beyond the four ps and the customer. J Public Policy Mark.

