# Supplementary material for: Breastfeeding, first-food systems and corporate power: a case study on the market and political practices of the transnational baby food industry in Brazil
Source: Global Health. 2024 Feb 6;20:12. doi: 10.1186/s12992-024-01016-0 (PMC10848415; doi:10.1186/s12992-024-01016-0)
Supplement: Supplementary file 1 — Supplementary Material 1 [file 12992_2024_1016_MOESM1_ESM.docx]

**Table S1.** Key actors influencing Brazil’s first-food system

| Acronym | Name | Sub-type | Year established | Website |
| --- | --- | --- | --- | --- |
| ADP | Amigas do peito | Civil Society | 1980 | <http://www.amigasdopeito.org.br/index.php/quem-somos/onde-estamos> |
| ANDI | Comunicação e Direitos | Civil Society | 1997 | <http://www.andi.org.br/jac/o-projeto> |
| WABA | World Alliance for Breastfeeding Action | Civil society | 1991 | <https://waba.org.my/news/portstrut.htm> |
| RBLH | Rede Global de Banco de Leite Humano | Civil Society | 1998 | <https://rblh.fiocruz.br/pagina-inicial-rede-blh> |
| ABRANDI | Associaçao Brasileira de Nutriçao Materno Infantil | Civil Society |  | <https://abranmi.com/sobre/> |
| FMCS | Fundacao Maria Cecilia Souto | Civil Society | 2007 | <https://www.fmcsv.org.br/pt-BR/> |
| ALIANÇA | Aliança Pela Alimentacao Adecuada e Saudável | Civil Society | 2017 | <http://alimentacaosaudavel.org.br/categoria/blog/> |
| RNPI | Rede Nacional Primeira Infancia | Civil Society | 2007 | <http://primeirainfancia.org.br/quem-somos/> |
| RedBLH | Rede Brasileira de Bancos de Leite Humano | Civil Society | 1998 | <http://www.redeblh.fiocruz.br/cgi/cgilua.exe/sys/start.htm?tpl=home> |
| AMO | Aleitamento | Civil Society | 1996 | <http://www.aleitamento.com/> |
| ACT | ACT Promoção da Saúde | Civil Society | 2006 | <http://actbr.org.br/> |
| IDEC | Instituto Brasileiro de Defesa do Consumidor | Civil Society | 1987 | <https://idec.org.br/> |
| IBFAN | IBFAN | Civil Society | 1983 | <http://www.ibfan.org.br/site/> |
| SPAPS | Ministério da Saúde - Secretaria de Atenção Primária à Saúde | Government | 2019 | [http://aps.saude.Government.br/](http://aps.saude.gov.br/) |
| CGAN | Ministério da Saúde - Coordenação Geral de Alimentação e Nutrição | Government |  | No webpage found |
| CGSCAM | Ministério da Saúde - Coordenação Geral de Aleitamento Materno e Saúde da Criança | Government |  | [https://saude.Government.br/saude-de-a-z/crianca](https://saude.gov.br/saude-de-a-z/crianca) |
| Anvisa | Agenda Nacional de Vigilancia Sanitaria | Government | 1999 | [http://portal.anvisa.Government.br/](http://portal.anvisa.gov.br/) |
| FNDCA | Fórum Nacional dos Direitos da Criança e do Adolescente | Government |  | [https://www.direitosdacrianca.Government.br/temas/redes_teste/forum-dca/](https://www.direitosdacrianca.gov.br/temas/redes_teste/forum-dca/) |
| CONANDA | Ministério da Mulher, da Família e dos Direitos Humanos - Conselho Nacional dos Direitos da Criança e do Adolescente | Government | 1991 | [https://www.mdh.Government.br/informacao-ao-cidadao/participacao-social/conselho-nacional-dos-direitos-da-crianca-e-do-adolescente-conanda](https://www.mdh.gov.br/informacao-ao-cidadao/participacao-social/conselho-nacional-dos-direitos-da-crianca-e-do-adolescente-conanda) |
| CNAM | Comitê Nacional de Aleitamento Materno | Government |  | Website not found |
| FPDPI | Frente Parlamentar pela Defesa da Primeira Infância | Government | 1987 | <https://frentedaprimeirainfancia.com.br/> |
| RNPI | Rede Nacional Primera Infançia | Government/Civil society | 2007 | <http://primeirainfancia.org.br/> |
| NES | Nestlé | Industry | 1875 | <https://www.nestle.com.br/> |
| FNB | Fundação Nestlé Brasil | Industry | 1987 | <https://www.nestle.com.br/proposito/fundacao-nestle-brasil> |
| DAN | Danone | Industry | 1970 | <http://corporate.danone.com.br/> |
| MJN | Mead Johnson | Industry | 1891 | <http://meadjohnson.com.br/> |
| CHO | Chicco | Industry | 1958 | <https://www.chicco.com.br/> |
| NML | Nutrimental | Industry | 1968 | <http://nutrimental.com.br/nossas-marcas/nutrilon/> |
| ILSI | International Life Sciences Institute | Industry | 1990 | <https://ilsibrasil.org/> |
| ABRAPUR | Associaçao Brasileira de Productos Infantis | Industry | 1995 | <http://www.abrapur.com.br/> |
| CONAR | Código Brasileiro de Autorregulamentação Publicitária | Industry | 1965 | <http://www.conar.org.br/> |
| CNA | Confederação da Agricultura e Pecuária do Brasil | Industry | 1951 | <https://www.cnabrasil.org.br/> |
| ABMRA | Associacao Brasilera de marketing rural e agronegocio | Industry | 1979 | <http://abmra.org.br/> |
| ABIA | Associação Brasilera da Industria Alimentos | Industry | 1963 | [https://www.abia.org.br](https://www.abia.org.br/) |
| ABIAD | Associação Brasileira da Indústria de Alimentos para Fins Especiais e Congêneres | Industry | 1986 | <http://abiad.org.br/pb/> |
| CFN | Conselho Federal de nutricionistas | Industry | 1978 | <https://www.cfn.org.br/> |
| UNICEFBR | UNICEF Brasil | International organization | 1950 | <https://www.unicef.org/brazil/> |
| OPAS | Organização Pan Americana da Saúde | International organization | 1902 | <https://www.paho.org/bra/> |
| ABRASCO | Associaçao Brasileira de Saude Coletiva | Professional association | 1979 | <https://www.abrasco.org.br/site/> |
| SBP | Sociedade Brasileira de Pediatria | Professional association | 1910 | <https://www.sbp.com.br/> |
| AMB | Associação Médica Brasileira | Professional association | 1951 | <https://amb.org.br/> |
| ABRAN | Associação Brasileira de Nutrologia | Professional association | 1973 | <https://abran.org.br/> |
| ABENFO | Associação Brasileira de Obstetrizes e Enfermeiros Obstetras | Professional association | 1954 | <http://abenfo.wixsite.com/meusite> |
| FEBRASCP | Federação Brasilera das Associaçõnes de Ginecologia e obstetricias | Professional association | 1959 | <https://www.febrasgo.org.br/pt/institucional/a-febrasgo> |
| SBAN | Associaçao Brasileira de Alimentação e Nutrição | Professional association | 1985 | <http://www.sban.org.br/historico.aspx> |
| FIOCRUZ | Fundação Oswaldo Cruz | Research organization | 1900 | <https://portal.fiocruz.br/> |
